# Evaluation of anticataract potential of Triphala in selenite-induced cataract: *In vitro* and *in vivo* studies

**DOI:** 10.4103/0975-9476.74425

**Published:** 2010

**Authors:** Suresh Kumar Gupta, V. Kalaiselvan, Sushma Srivastava, Shyam S. Agrawal, Rohit Saxena

**Affiliations:** *Delhi Institute of Pharmaceutical Sciences and Research, New Delhi, India*; 1 *All India Institute of Medical Sciences, New Delhi, India*

**Keywords:** Anticataract, antioxidant, glutathione, malondialdehyde, selenite, superoxide dismutase, triphala

## Abstract

Triphala (TP) is composed of *Emblica officinalis, Terminalia chebula*, and *Terminalia belerica*. The present study was undertaken to evaluate its anticataract potential *in vitro* and *in vivo* in a selenite-induced experimental model of cataract. *In vitro* enucleated rat lenses were maintained in organ culture containing Dulbecco’s Modified Eagles Medium alone or with the addition of 100*μ*M selenite. These served as the normal and control groups, respectively. In the test group, the medium was supplemented with selenite and different concentrations of TP aqueous extract. The lenses were incubated for 24 h at 37°C. After incubation, the lenses were processed to estimate reduced glutathione (GSH), lipid peroxidation product, and antioxidant enzymes. *In vivo* selenite cataract was induced in 9-day-old rat pups by subcutaneous injection of sodium selenite (25 *μ*mole/kg body weight). The test groups received 25, 50, and 75 mg/kg of TP intraperitoneally 4 h before the selenite challenge. At the end of the study period, the rats’ eyes were examined by slit-lamp. TP significantly (*P* < 0.01) restored GSH and decreased malondialdehyde levels. A significant restoration in the activities of antioxidant enzymes such as superoxide dismutase (*P* < 0.05), catalase (*P* < 0.05), glutathione peroxidase (*P* < 0.05), and glutathione-s-transferase (*P* < 0.005) was observed in the TP-supplemented group compared to controls. *In vivo* TF 25mg/kg developed only 20% nuclear cataract as compared to 100% in control. TP prevents or retards experimental selenite-induced cataract. This effect may be due to antioxidant activity. Further studies are warranted to explore its role in human cataract.

## INTRODUCTION

Cataract is the major cause of blindness responsible for 50% of the global incidence.[1] Pharmacological intervention that prevents or slows progression of cataractogenesis has a significant health impact. Our earlier studies screened natural antioxidants and herbal drugs and reported their potential anticataract activity.[2–5]

Triphala is one of the commonest and cheapest of herbal preparations available in India. It is composed of equal parts of three most valuable herbs, Indian Gooseberry (*Emblica officinalis*), Chebulic Myrobalan (*Terminalia chebula*), and Beleric Myrobalan (*Terminalia belerica*).[6,7] These drugs have been evaluated for their comparative antidiabetic and antioxidant activities.[8] Triphala is well documented as a rejuvenator and antioxidant.[9–11] It has been scientifically validated for its anti-inflammatory[12] and hypolipidemic effects.[13,14] In recent years, a number of research studies have confirmed its potential in the treatment of various types of cancer.[6]

Triphala preparations from different manufacturers have been marketed for the treatment of cataract. However no scientific evidence is available supporting the use of Triphala as a therapeutic agent for eye diseases. The aim of the present study was to assess the potential of Triphala aqueous extract in selenite-induced experimental cataractogenesis in a rat pups model, and its effect on the antioxidant enzymes superoxide dismutase (SOD), catalase (CAT), glutathione-s-transferase (GST), glutathione peroxidase (GPX), glutathione (GSH), and levels of lipid peroxidation determined *in vitro* in rat lens culture.

## MATERIALS AND METHODS

### Drugs and chemicals

These were obtained from the following sources: chemicals required for the enzyme assay from Sigma Chemical Co., USA; sodium selenite, oxidative stress inducing agent, from Central Drug House (P) Ltd. New Delhi; Triphala extract from Promed Exports Pvt Ltd., New Delhi.

### Animals

Wistar rat pups of either sex (10–15g) were procured from the animal house, Delhi Institute of Pharmaceutical Sciences and Research, after obtaining approval from our institutional Animal Ethics Committee. Animals in the study were treated in accordance with the institutional guidelines and Association for Research in Vision and Ophthalmology statement for the use of animals in research. Mothers and suckling pups were left to acclimatize undisturbed for 4 days before the experiment.

### Preparation of the Triphala extract

One hundred grams Triphala powder was boiled in 1000 ml distilled water until the volume had reduced to one-quarter of the original. The extract was cooled, centrifuged in a cold centrifuge, and the supernatant collected and lyophilized.[4] An 18.4%w/w yield was obtained and used for the *in vitro* and *in vivo* studies.

### Preliminary phytochemical analysis

The extract was screened for the presence of tannins, flavonoids, and phenolic compounds using the methods described by Tona.[15] High Performance Thin Layer Chromatography (HPTLC; Camag, Japan) was used to identify phenolic compounds in Triphala extract. Precisely 1 gram of extract was dissolved in 25ml of methanol; after warming the content with shaking the solution was filtered through Whatman filter paper No.1 and the filtrate collected. The solvent was evaporated over a water bath to obtain the residue, which was dissolved in 50ml methanol. Ten microliter of the resulting sample was applied using Camag Linomat-5 on a precoated silica gel 60 F254 TLC plate on aluminum sheet of uniform thickness (0.2mm). The plate was developed in a solvent system consisting of chloroform–ethyl acetate–formic acid (4:5:1). It was then sprayed with vanillin in sulfuric acid and scanned at UV-254 nm using a Camag Scanner-3.

### *In vitro* studies with the lowest effective concentration of Triphala

The rats were anesthetized with ether. The anterior portion of both eyes of each rat was removed by cutting just posterior to the limbus using a coaxial operating microscope for magnification, and stainless-steel surgical equipment. The lens was removed (without disturbing its capsular integrity) after cutting suspensory ligaments; care was taken to avoid contamination from neighboring tissues and environmental sources. Freshly dissected lenses were rolled in filter paper to remove all adherent vitreous fluid. Each isolated lens was placed in a Falcon plastic culture plate (24-well) containing 2ml of Dulbecco’s Modified Eagles Medium (DMEM) supplemented with 20% fetal bovine serum, 100μg/ml of streptomycin, and 100 IU/ml penicillin. The lenses were incubated at 37°C under 90% moisture, 95% air, and 5% CO_2_ gas atmosphere for 2 h. Damaged lenses that developed artificial opacities were discarded, and only transparent lenses were taken for subsequent *in vitro* experiments.

### Selenite-induced oxidative stress

Transparent cultured lenses were randomly divided into normal, control, and three treatment groups each comprising six lenses. Normal lenses were incubated in DMEM alone, while control group lenses were incubated in DMEM supplemented with 100 μM sodium selenite. The medium for the treated groups was additionally supplemented with three different concentrations of Triphala (400, 800, and 1200μg/ml were selected after conducting a pilot study) along with selenite. All the lenses in different groups were incubated for 24 h under these conditions. Post-incubation, the lenses were examined for the presence of any opacity, and photo documentation carried out. Thereafter, lenses were washed, weighed, and processed for estimation of biochemical parameters. Each lens was homogenized in 1ml of 0.1 M-phosphate buffer (pH 7). The homogenate was divided into two equal parts. One part was used for the estimation of GSH and the other for malondialdehyde.

### Estimation of glutathione[16]

The homogenate was centrifuged at 5000 rpm for 15 min at 4°C. To the supernatant, 0.5ml of 10% trichloroacetic acid was added and recentrifuged. The protein- free supernatant thus obtained was reacted with 4ml of 0.3 M of Na_2_HPO_4_ (pH 8.0) and 0.5ml of 0.04% (w/v) 5,5’-dithiobis-2-nitrobenzoic acid. The absorbance of the resulting yellow color was measured in a spectrophotometer at 412 nm. A parallel standard was also maintained.

### Estimation of malondialdehyde[17]

The homogenate was mixed with 0.15M KCl and centrifuged at 10,000 rpm for 10 min. 0.2ml of the supernatant was reacted with 0.2 ml of 8.1% of SDS, 1.5ml of 20% acetic acid (pH 3.5), and 1.5ml of TBA. All the samples were heated in a boiling water-bath for 60 min. After cooling, 5ml of *n*-butanol:pyridine mixture was added to each sample. The solution was shaken vigorously in a vortex and centrifuged at 5,000 rpm for 10 min. The organic ayer was separated and absorbance observed in the spectrophotometer at 515 nm. Simultaneously various amounts of 1,1’3, 3’-tetra ethoxy propane (TEP) were used to obtain standard curves for calculation of unknown malondialdehyde (MDA) in the samples.

### Enzyme assay

A separate set of experiments was conducted under the same experimental conditions as described above. After incubation for 24 h, lenses of each group were processed to measure activities of the enzymes superoxide dismutase (SOD), catalase (CAT), glutathione peroxidase (GPX), and glutathione-*S*-transferase (GST). 10% (w/v) lens homogenate was prepared in 50 mM of phosphate buffer (pH 7.0) after centrifuging at 5000 rpm for 15 min at 4°C, and the supernatant used for measurement of enzyme activities.

### Superoxide dismutase[18]

The ability of the enzyme to inhibit the oxidation of epinephrine was monitored spectrophotometrically at 480 nm.[10] One unit of SOD activity is defined as the amount of enzyme required to produce 50% inhibition of epinephrine auto-oxidation.

### Catalase[19]

The enzyme activity was measured at 240 nm tracking the decomposition of H_2_O_2_ spectrophotometrically at 240 nm. One unit of CAT activity is defined as nmol H_2_O_2_ decomposed per min/mg protein.

### Glutathione peroxidase[20]

Activity was monitored at 340 nm. One unit of enzyme activity is defined as 1 nmol of NADPH used per minute at 37°C.

### Glutathione-s-transferase[21]

The conjugation of GSH with 1 chloro, 2-4 dinitro benzene (CDNB), a hydrophilic substrate, was observed spectrophotometrically at 340 nm to measure the activity of GST. One unit of GST is defined as the amount of enzyme required to conjugate 1 μmol of CDNB with GSH/min. Protein content in each sample was estimated.[22]

### *In vivo* studies

*Selenite cataract*. Nine-day-old Wistar rat pups were divided into control and treated groups. In each group (*n*=10), pups of the same litter were housed with the mother. Acute stress was produced by a single subcutaneous injection of 25 μmol sodium selenite per kg body weight into all the pups in all groups on the 9th postpartum day. Triphala aqueous extract at doses of 25, 50, and 75mg/kg body weight was injected intraperitoneally to the pups in the treated groups on postpartum days 9–14. Incidence of cataract was observed through a slit lamp after dilating the pupil with 1% tropicamide on the 18th postpartum day when the pups first opened their eyes.

### Statistical analysis

All data were expressed as mean ± SD. The groups were compared by one-way ANOVA using post-hoc Dunnett’s test and Chi Square test for *in vivo*.

## RESULTS

### Preliminary phytochemical screening

**Figure 1:**
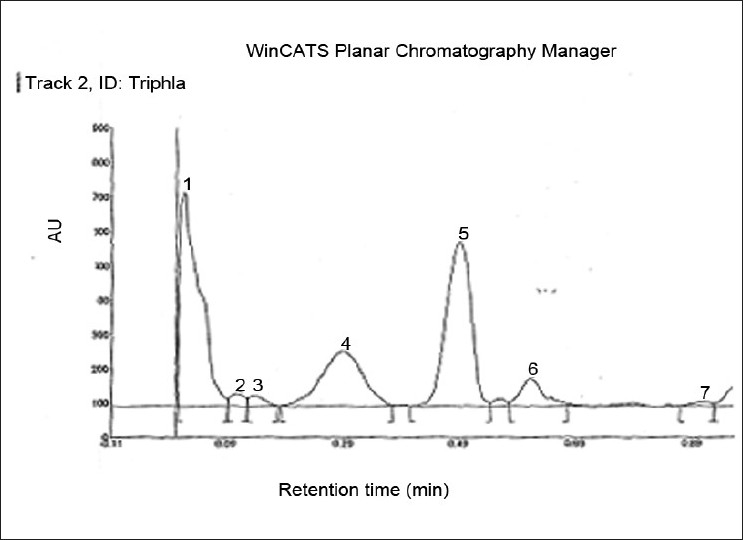
HPTLC profile of the phenolic compounds of methanolic extract of Triphala, Two major peaks and five minor peaks separated from the methanolic extract. The peaks labeled 1, 4 and 5 accounted for 35.20, 19.05 and 34.37% of the total area respectively

Preliminary phytochemical screening detected the presence of tannins, flavonoids and phenolic compounds in Triphala aqueous extracts. Two major peaks and five small peaks separated from the Triphala methanolic extract of the, the two labeled 1 and 5 accounting for 35.2% and 34.3%, respectively, of their total area [Figure 1].

### Effect on lens morphology

**Figure 2:**
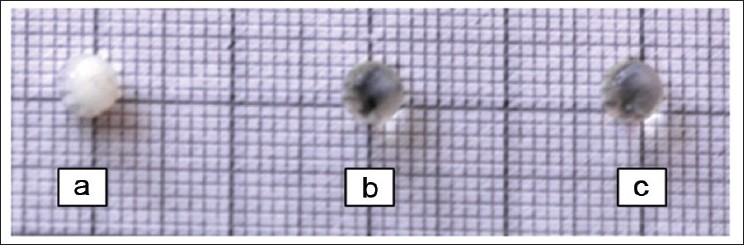
Effect of Triphala on morphology of lens, (a) Control (DMEM+100μM of sodium selenite), (b) Normal (DMEM only) remains transparent after 24 h incubation, (c) Treated (DMEM+100μM of sodium selenite+Triphala 800 μg/ml)

All the lenses in DMEM alone were transparent. However, lenses after 24 h of incubation in the presence of sodium selenite developed nuclear opacity. Incorporation of Triphala in the medium of the treated group offered significant protection from selenite stress. Only 33% of the lenses in the treated group showed cortical opacity (*P* < 0.01) [Figure 2].

### Effect on glutathione and malondialdehyde (oxidative stress markers)

**Figure 3:**
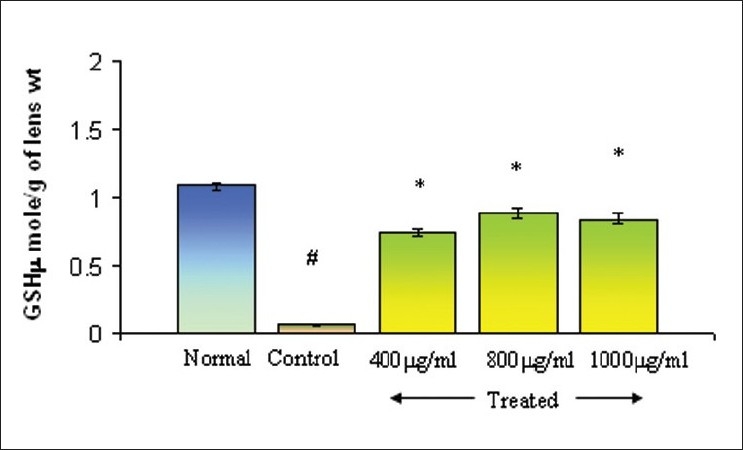
Effect of Triphala on GSH levels in selenite-induced oxidative stress *in vitro*, Normal: DMEM, Control: DMEM+100μM of Sodium selenite, Treated: DMEM+100μM of Sodium selenite + Triphala. Incubation period 24 h. Values are mean±SD. *P<0.01 (control vs treated) and #*P* <0.005 (control vs normal) as compared to contril. n=6.

**Figure 4:**
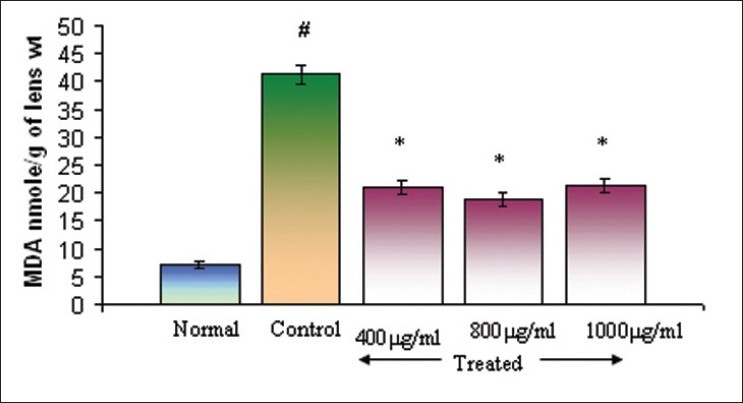
Effect of Triphala on MDA levels in selenite-induced oxidative stress *in vitro*, Normal: DMEM, Control: DMEM+100μM of Sodium selenite, Treated: DMEM+100μM of Sodium selenite + Triphala. Incubation period 24 h. Values are mean±SD. *P<0.01 and #*P* <0.05 as compared to control. n=6.

Changes in GSH and MDA levels were evaluated in lenses cultured in the presence of sodium selenite (100 μM) using different concentrations of Triphala. GSH levels in different groups are presented in Figure 3. GSH level in the normal group was estimated to be 1.08 ± 0.02 μmol/g of lens, whereas the GSH content of lenses in the control group was found to be 0.07 ± 0.007 μmol/g. Incorporation of Triphala into the culture medium significantly restored GSH levels. Values of 0.74 ± 0.02, 0.88 ± 0.03, and 0.84 ± 0.02 were obtained at concentrations of 400, 800, and 1200μg/ml, respectively. MDA levels in controls in the presence of sodium selenite produced the significant increase in lipid peroxidation of 41.33 ± 1.83 nmol/g of lens. However, in the presence of Triphala, lipid peroxidation was significantly reduced and MDA values were respectively found to be 20.93 ± 1.25, 18.82 ± 1.22, and 21.20 ± 1.13 nmol/g of lens at concentrations of 400, 800, and 1200 μg/ml [Figure 4].

### Effect on antioxidant enzyme activities

Selected key enzymes in the oxidative defense system were studied in presence of sodium selenite, the activities of SOD, GPX, CAT, and GST were reduced significantly in control group lenses in comparison to that of the normal lenses. However, positive modulation of antioxidant enzymes activity was observed at Triphala concentrations of 800 μg/ml [Table 1].

**Table 1:** Effect of Triphala on antioxidant enzyme levels in rat lens

Groups	SOD	CAT	GPX	GST

	(IU/mg protein)						
Normal	3.11,± 0.59	1.50 ± 0.01	10.87 ± 0.99	3.23 ± 0.57	
Control	0.86 ± 0.09^a^	0.18 ± 0.02^a^	3.61 ± 0.70^b^	1.90 ± 0.12^b^
Treated	1.26 ± 0.12^c^	0.61 ± 0.04^c^	6.82 ± 0.41^c^	3.01 ± 0.28^d^

Normal: DMEM, control: DMEM + sodium selenite, treated: DMEM+ sodium selenite + Triphala 800 μg/ml. Incubation period 24 h. Values are mean ± SD.

a *P* < 0.05

b *P* < 0.005 (control vs normal),

c *P* < 0.05

d *P* < 0.005 (treated vs control); n=6.

### Effect on selenite cataract: *In vivo*

**Figure 5:**
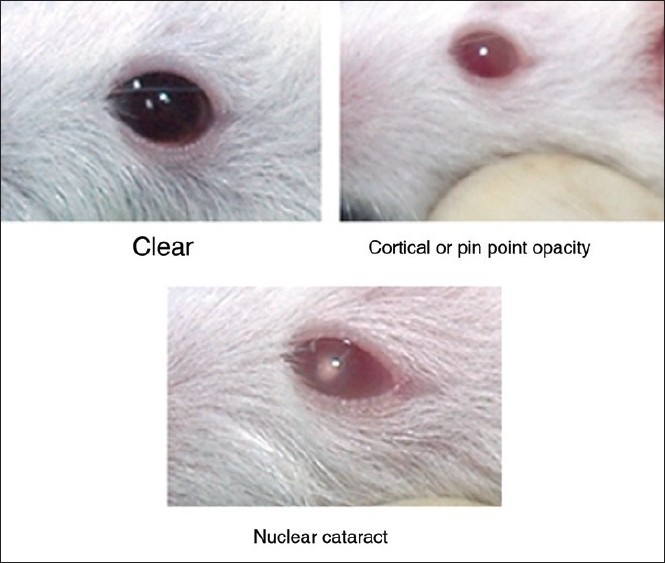
Different grades of selenite cataract in rat pups

**Figure 6:**
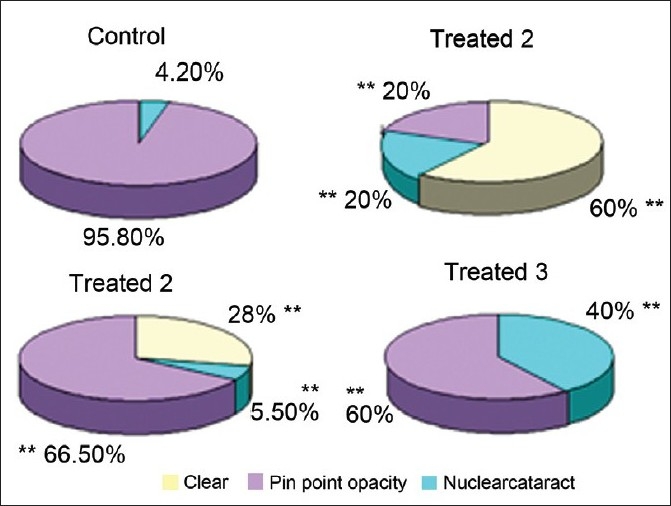
Effect of Triphala on selenite cataract in rat pups, Control: Sodium selenite Treated 1: Sodium selenite-Triphala 25 mg/kg, Treated 2: Sodium selenite-Triphaia 50mg/kg and Treated 3: Sodium selenite-Triphala 75 mg/kg, **P<0.005 (Control vs treated) n**12.

Different grades of selenite cataracts are demonstrated in Figure 5. Subcutaneous injection of μmol concentrations of sodium selenite led to the development of 100% nuclear opacities in the eyes of the control group on postnatal day 18. Of these, 4.2% of the eyes developed pinpoint opacity and 95.8% developed nuclear cataract. In contrast Triphala 25mg/kg led to 60% of the eyes being clear, 20% with pinpoint opacity, and only 20% developing nuclear cataract. Triphala 50 mg/kg resulted in 28% of the eyes being clear, 66.5% with nuclear cataract, and 5.5% with pinpoint opacity. In case of Triphala 100mg/kg, none of the eyes were clear whereas 60% nuclear cataract and 40% pinpoint opacity [Figure 6].

## DISCUSSION

Triphala is currently used for treatment of eye diseases, especially cataract, based on empirical knowledge, and to this date no scientific documentation has been published. Cataract is a major cause of blindness leading to 50% of blindness globally.[23] Surgically, cataractous lens can be replaced by artificial lens; however, epidemiologically the problem is not reduced owing to expense and post-operational complications of surgery.[24] For these reasons, the present study focused on a pharmacological intervention for cataract, in particular, scientific validation of Triphala in the treatment of cataract.

The selenite experimental model was selected because of its rapid, effective, and reproducible cataract formation. As a model for senile nuclear cataract, it has been extensively characterized histologically and biochemically.[25] It shows a number of general similarities to human cataract in addition to vesicle formation, such as increased calcium, insoluble protein, proteolysis, and decreased glutathione levels.[26] Selenite damages lens epithelial cells by acting as a sulfhydryl oxidant followed by opening of calcium channel or inhibition of calcium ATPase. This leads to accumulation of calcium, activation of calpain, proteolysis of beta crystalline polypeptides that finally form insoluble pellets which scatter light. Although the rate of opacification in the selenite model is much more rapid than in human cataract, it has many general similarities to human cataract such as increased calcium, protein aggregation, decreased water-soluble proteins, and reduced glutathione levels.[27] Various herbal and natural drugs have been explored for their potential against cataract in selenite-induced experimental models.[28–31]

The present study evaluated the anticataract potential of Triphala against sodium selenite-induced cataract *in vitro* and *in vivo* models. It found a correlation between *in vitro* and *in vivo* anticataract potential of Triphala in a selenite-induced oxidative stress model. *In vitro* oxidative stress was induced by adding the selenite to the medium to which the rat lenses were exposed, and antioxidant parameters and enzymes were estimated. *In vivo*, oxidative stress was induced by injecting sodium selenite into the rat pups, and the Triphala’s protective effect (nuclear cataract) was evaluated. Sodium selenite 100μM was chosen for the present study since this level had been used in earlier studies.[28,32] Exposure of rat lenses to the selenite in primary culture damaged cell morphology, and its overall antioxidant status such as GSH and antioxidant enzymes. Lens levels of GSH were significantly reduced. GSH maintains proteins in their reduced form by means of its sulfhydryl group. Reduced levels of GSH were observed in cataractous lenses, offering an explanation for their observed pathology.[33,34] In the present investigation, we found that Triphala significantly restored the level of GSH in 24 h cultured rat lens. The present findings corroborate earlier studies where Triphala significantly restored depleted GSH levels in intestinal mucosa of methotrexate-treated rats.[34] Triphala restored GSH levels in a concentration-dependent manner with optimal activity occurring at a dose of 800μg/ml, activity being constant up to 100μg/ml. With further increase in the drug concentration, a gradual decline in GSH activity was observed.

In eye lenses, reactive oxygen species attack biological molecules, including DNA, protein, and phospholipids leading to lipid peroxidation and depletion of the antioxidant enzymes, SOD, GST, GPX, and catalase, resulting in further oxidative stress.[35] In accordance with previous findings, our study observed high lipid peroxidation with concomitant decreases in lens antioxidant enzymes, under selenite stress. Triphala extract was observed to exhibit anticataract effect as demonstrated by enhanced activities of antioxidant enzymes, GSH, and diminished amount of lipid peroxide against selenite-induced oxidative stress.

Selenite-induced cataract *in vivo* (20–30 nmol/kg) causes nuclear opacity through the calpain proteolysis of lens proteins. Selenite is a strong sulfhydryl oxidant and is considered a valid model for cataracts caused by oxidative stress.[36] Similar to human senile cataract, this type of cataract is accompanied by a decrease in activities of antioxidant enzymes such as SOD and GPX.[29] A previous study showed that flavonoids, with antioxidant properties, can prevent oxidative damage and slow experimental selenite cataract progression.[37] Triphala has been reported to be a rich source of vitamin C and flavanoids.[38] The present investigation, confirmed that intraperitoneal administration of Triphala 25mg/kg into the rat pups could prevent selenite-induced cataract formation; only 20% eyes developed nuclear cataract in the Triphala-treated group, compared to 100% in selenite-injected group.

## CONCLUSIONS

The present findings show that Triphala prevents selenite-induced experimental cataractogenesis *in vitro* and *in vivo*. Further studies are in progress to establish mechanisms of action at the molecular level.
